# Epithelial derived-matrix metalloproteinase (MMP9) exhibits a novel defensive role of tumor suppressor in colitis associated cancer by activating MMP9-Notch1-ARF-p53 axis

**DOI:** 10.18632/oncotarget.13406

**Published:** 2016-11-16

**Authors:** Lewins Walter, Adani Pujada, Noopur Bhatnagar, Agnieszka B Bialkowska, Vincent W. Yang, Hamed Laroui, Pallavi Garg

**Affiliations:** ^1^ Institute for Biomedical Sciences, Georgia State University, Atlanta, GA, USA; ^2^ Department of Biology, Georgia State University, Atlanta, GA, USA; ^3^ Stony Brook University, Department of Medicine, Stony Brook, NY, USA; ^4^ Center for Diagnostics and Therapeutics, Department of Chemistry/Biology, Georgia State University, Atlanta, GA, USA

**Keywords:** MMP9, tumor suppressor, colitis associated cancer, Notch1, γH2AX

## Abstract

Colitis associated cancer (CAC) is chronic inflammation driven colon cancer, prevalent among individuals with Inflammatory Bowel Disease. Matrix-metalloproteinase (MMP9) is one of the essential regulators of extra cellular matrix components. We have shown that MMP9 is protective in CAC contrary to its inflammatory role in acute-colitis. Aim of our study is to identify the mechanism of the protective role of epithelial derived-MMP9 in CAC. We used homozygous transgenic mice constitutively-expressing MMP9 in colonic-epithelium (TgM9) and wild-type (WT) littermates for *in vivo* experiments. Stably-transfected HCT116 with/without MMP9, and mouse embryonic-fibroblasts (WT and MMP9^−/−^, MEFs) were used for *in vitro* experiments. TgM9 mice exhibited less tumor burden, increased apoptosis, and increased expressions of active-Notch1, p53, p21^WAF1/Cip1^, caspase-3 and cyclin E in CAC compared to WTs. These results were supported by MEFs data. HCT116-cells overexpressing MMP9 indicated decreased cell proliferation, S-phase cell-cycle arrest and less DNA damage compared to vector. MMP9^−/−^ mice showed attenuation of MMP9 was directly associated with p19ARF. Our study identifies the tumor suppressor role of epithelial derived-MMP9 in CAC via novel mechanistic pathway “MMP9-Notch1-ARF-p53 axis” regulating apoptosis, cell-cycle arrest and DNA damage implying, that MMP9 expression might be a natural/biological way to suppress colonic ulceration due to chronic inflammation.

## INTRODUCTION

The role of inflammation in cancer development has been indicated as early as in the 18^th^ century [1]. In the following years, an association between chronic inflammation and cancer has been strongly highlighted. Inflammation is a beneficial response to tissue damage and pathogenic challenges, though unregulated inflammation may transform into chronic, which induces malignant cell transformation in the tissue environment. Inflammatory bowel disease (IBD), which primarily includes ulcerative colitis (UC) and Crohn's disease (CD), involves inflammation of all or part of digestive tract. Patients with chronically active UC have significantly higher risk (as high as 50% depending on the population cohort) of colitis associated cancer (CAC) [2]. CAC risk increases with the duration of the disease and correlates positively with the severity of inflammation. CAC is a subtype of colorectal cancer (CRC) yet uniquely different from it [3]. CAC progresses through ‘dysplasia-carcinoma axis’ while CRC progression is through ‘adenoma-carcinoma axis’ [4]. In CAC, *p53* mutations are the first ones to be detected followed, by *KRAS* and *APC* mutations respectively. On the other hand in CRC *APC* mutations are the first ones to be identified followed by *KRAS* mutations and *p53* mutations respectively [3, 4]. It is known that inflammatory responses dissect oncogenic signaling pathways such as apoptosis, proliferation, angiogenesis etc.

In this context, its worth to mention the indispensable role played by a large family of proteolytic enzymes called proteinases [5]. Among the wide variety of proteinases, the matrix metalloproteinases (MMPs) are the zinc dependent endo-proteinases that are highly active during inflammation, and play an important role in ulceration and tissue remodeling [3–6]. MMPs are widely distributed, however their regulation is complex and controlled at various levels such as transcriptional, post translational and by endogenous MMP inhibitors [7]. MMPs are importantly linked to a wide variety of functions other than remodeling the extra cellular matrix (ECM) components [8]. They are involved in regulating cell–cell and cell–matrix signaling via activation of cytokines and growth factors. Being actively secreted proteinases, they have the ability to alter cell surface receptors and junctional proteins which tightly regulate inflammation and carcinogenesis. In recent years, studies to identify the molecular basis and pathophysiology of CAC have been attempted. However, the precise role of MMPs in mediating CAC is still unexplored except few sporadic studies [3, 9, 10–11].

Among the 25 known mammalian MMPs [12], MMP9 is unique as its protein expression and activity is undetectable in most healthy adult tissues including the intestine and colon but is highly expressed in a variety of inflammatory states. We have shown that epithelial cell-derived MMP9 mediates tissue damage during colitis [13–15]. We have also shown that despite being a mediator of acute colitis, MMP9 plays an opposite but protective role in the development of CAC [11–10]. Aim of the present study is to determine if epithelial derived-MMP9 is responsible for this contrasting but defensive role of tumor suppressor in CAC. We also sought to determine the underlying the molecular mechanism by using MMP9 transgenic mice, Tg-villin-MMP9 (TgM9) that constitutively expresses MMP9 in the colonic epithelium under villin promoter.

## RESULTS

### Constitutive expression of MMP9 in colonic epithelium exhibited resistance to CAC

**Figure 1:**
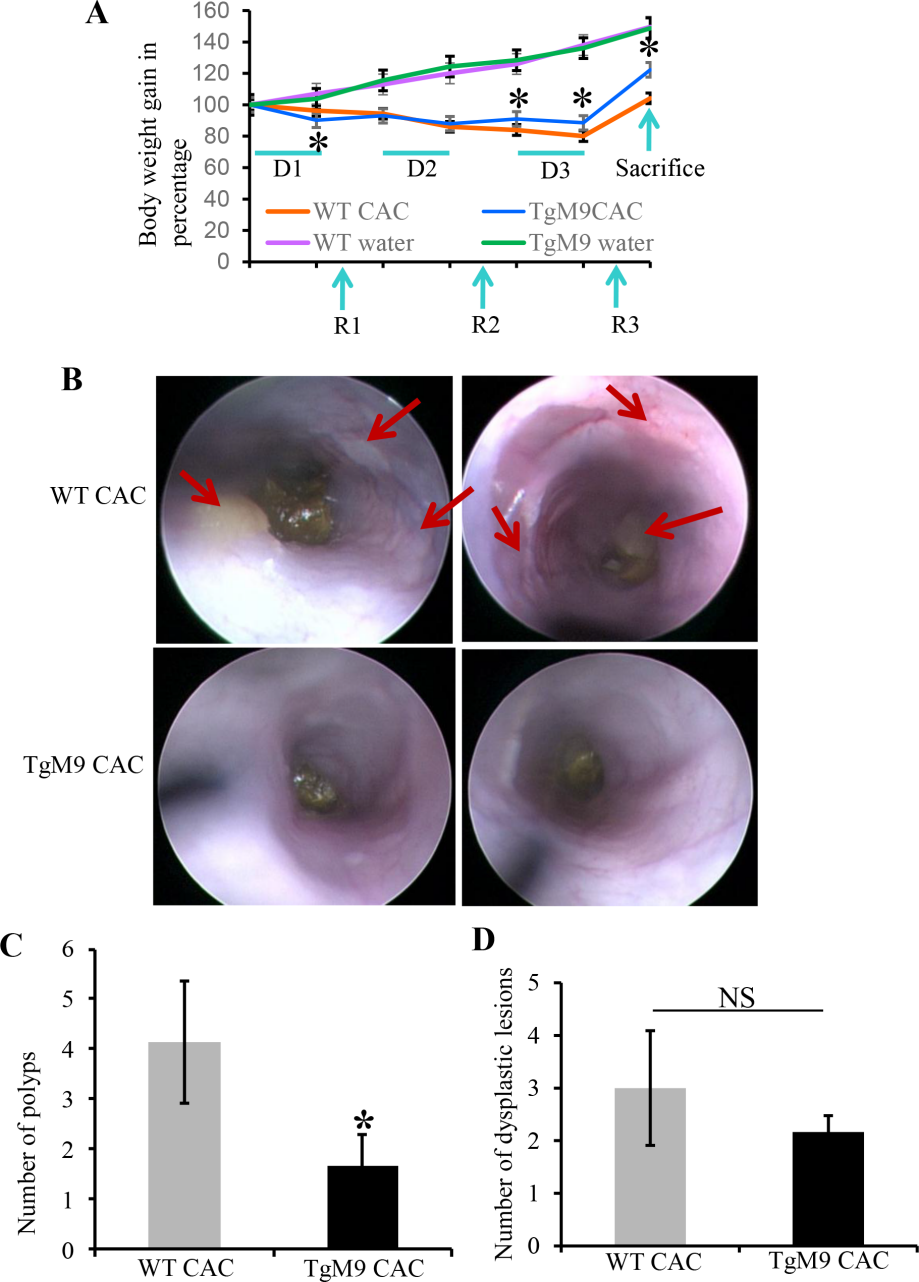
Constitutive expression of MMP9 in colonic epithelium exhibited resistance to CAC (**A**) The line graph representation of change in body weight of TgM9 mice (blue line) and WT mice (orange line) during three cycles of DSS (D1, D2 and D3) followed by three recovery cycles (R1, R2 and R3). TgM9 mice (green line) and WT mice (red line) without CAC induction shows the body weight change. (**B**) The top panel shows representative colonoscopy images from two different WT mice and bottom panel shows representative colonoscopy images from two different TgM9 mice in CAC. Red arrows indicate flat polyps a characteristic feature of CAC polyps. (**C**) The bar graph presentation of number of polyps among TgM9 mice (blue bar) and WTs (grey bar) in CAC. (**D**) The bar graph presentation of dysplastic lesions count among TgM9 mice (blue bar) and WTs (grey bar) in CAC, NS means non-significant. Each bar represents mean ± S.E., *p < 0.05.

Both TgM9 and their wild type (WT) littermates were induced with CAC as described in ‘Materials and Methods’ section and were sacrificed after 85 days. Both groups of mice were monitored for weight loss during the entire length of the experiment. TgM9 mice showed significantly more weight loss compared to WT littermates at the end of 1st cycle of dextran sodium sulfate (DSS) (Figure 1A). However, at the end of 2nd cycle of DSS there were no significant changes in the body weight of TgM9 mice compared to WTs. On the other hand, TgM9 mice started displaying a significant weight gain at the end of 2^nd^ recovery cycle (Figure 1A). They also exhibited significantly lower body weight loss compared to WTs at the end of 3^rd^ cycle of DSS. This trend continued until the end point of the protocol i.e. till the day of sacrifice. TgM9 and WT mice exhibited a parallel and comparable increase in body weight without CAC as a control group (Figure 1A). Figure 1B shows the colonoscopy view indicating that thickening of mucosal layer due to inflammation and number of polyps (as shown by red arrows), were significantly lesser among TgM9 mice compared to WTs in CAC. Figure 1C is the bar graph presentation of tumor incidence among TgM9 supporting the colonoscopy data. There was significantly less number of polyps among TgM9 mice (1.7 ± 0.64) compared to WT littermates (4.1 ± 1.2) in CAC. In Figure 1D, TgM9 showed a trend of fewer dysplastic lesions (2.2 ± 0.31) compared to their WT littermates (3 ± 1.09) in CAC, however the difference was not significant. These data together indicates that TgM9 mice constitutively expressing MMP9 in colonic epithelium were protected compared to WTs in CAC.

### Constitutive expression of MMP9 in colonic epithelium was associated with lower histological score and apoptosis in CAC

**Figure 2:**
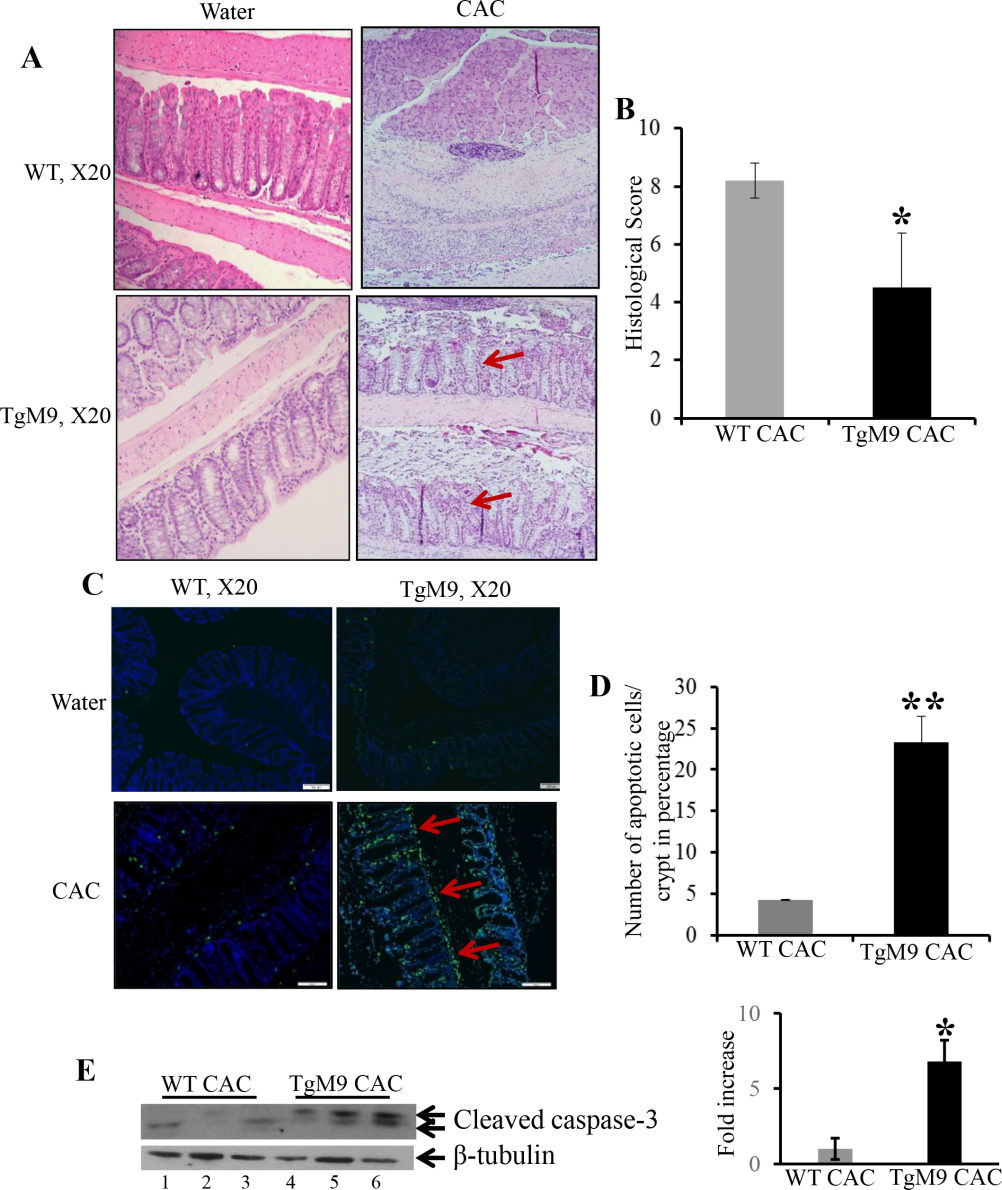
Constitutive expression of MMP9 in colonic epithelium was associated with lower histological score and apoptosis in CAC (**A**) H&E staining of Swiss rolls of the colons from TgM9 and WT mice in CAC indicating less damage to crypt architecture and fewer dysplastic lesions, as indicated by red arrows. Left panel represents TgM9 and WT mice exposed to water and right panel represents TgM9 and WT mice induced with CAC. (**B**) The bar graph presentation of histological score calculated on three parameters: infiltration of neutrophils, loss of crypt architecture and foci of ulceration of TgM9 mice (blue bar) and WTs (grey bar) in CAC. (**C**) The overlay images of greenish yellow nuclei of apoptotic cells as indicated by red arrow, magnification used was X20. (**D**) The bar graph representation of the quantification of apoptosis as the percentage of apoptotic nuclei/total number of cells per crypt among TgM9 mice (blue bar) and WTs (grey bar) in CAC, number of intact crypts counted were 12 for each mice. (**E**) WB of protein (25 μg/lane) from mucosal stripping of the TgM9 and WTs with CAC was performed and probed with anti-Caspase-3. The loading control for the blot was β-tubulin. Densitometry evaluations of the WB is represented by the adjacent bar graph. Each bar represents mean ± S.E., *p < 0.05 and **p < 0.005.

Figure 2A represents the Haematoxylin and Eosin (H&E) staining indicating decreased crypt architecture damage, lesser infiltration of neutrophils, fewer foci of ulceration and dysplastic lesions among TgM9 mice compared to WTs (as indicated by red arrows). Figure 2B is the bar graph presentation of the histological score based on the parameters described in ‘Materials and Methods’ section, indicating that TgM9 mice had significantly lower histological score (4.5 ± 1.9) compared to WTs (8.2 ± 0.6) in CAC. Abnormal apoptosis and/or irregular proliferation are the characteristic features of tumor cells. Terminal deoxynucleotidyl transferase-mediated dUTP nick end labeling (TUNEL) staining was performed to assess apoptosis in the colonic epithelium of TgM9 mice. Figure 2C represents the merged/overlay image of greenish yellow fluorescence indicating that there was a significant increase in apoptosis (as represented by red arrows at the apical surface of crypts) among the TgM9 mice compared to WTs in CAC. Figure 2D is the bar graph presentation of the quantification of apoptotic cells among TgM9 mice and WTs in CAC, indicating that TgM9 mice had significantly higher (23.29 ± 3.2) compared to WTs (4.24 ± 0.07) in CAC. We observed increased apoptosis through TUNEL staining hence we wanted to explore apoptosis associated proteins. The western blot (WB) analysis of apoptosis associated proteins showed significantly increased protein level of cleaved caspase-3 (Figure 2E) among TgM9 mice (lanes 4–6) compared to WTs (lanes 1–3). Compared to the quantification of the apoptotic cells as shown in Figure 2D, the WB in E showed higher cleaved caspase-3 activity. This can be explained by the fact that quantification was performed considering only the intact crypts not the dysplastic epithelium while WB represents the epithelial-mucosal cell lysates which included both dysplastic and the non-dysplastic epithelium. Together, these results indicate that in CAC epithelial derived-MMP9 expression maintained crypt architecture and was associated with fewer dysplasia and increased apoptosis compared to WTs.

### TgM9 mice exhibited altered protein expression of active Notch1, p53, p21 ^WAF1/Cip1^ and Cyclin E

**Figure 3:**
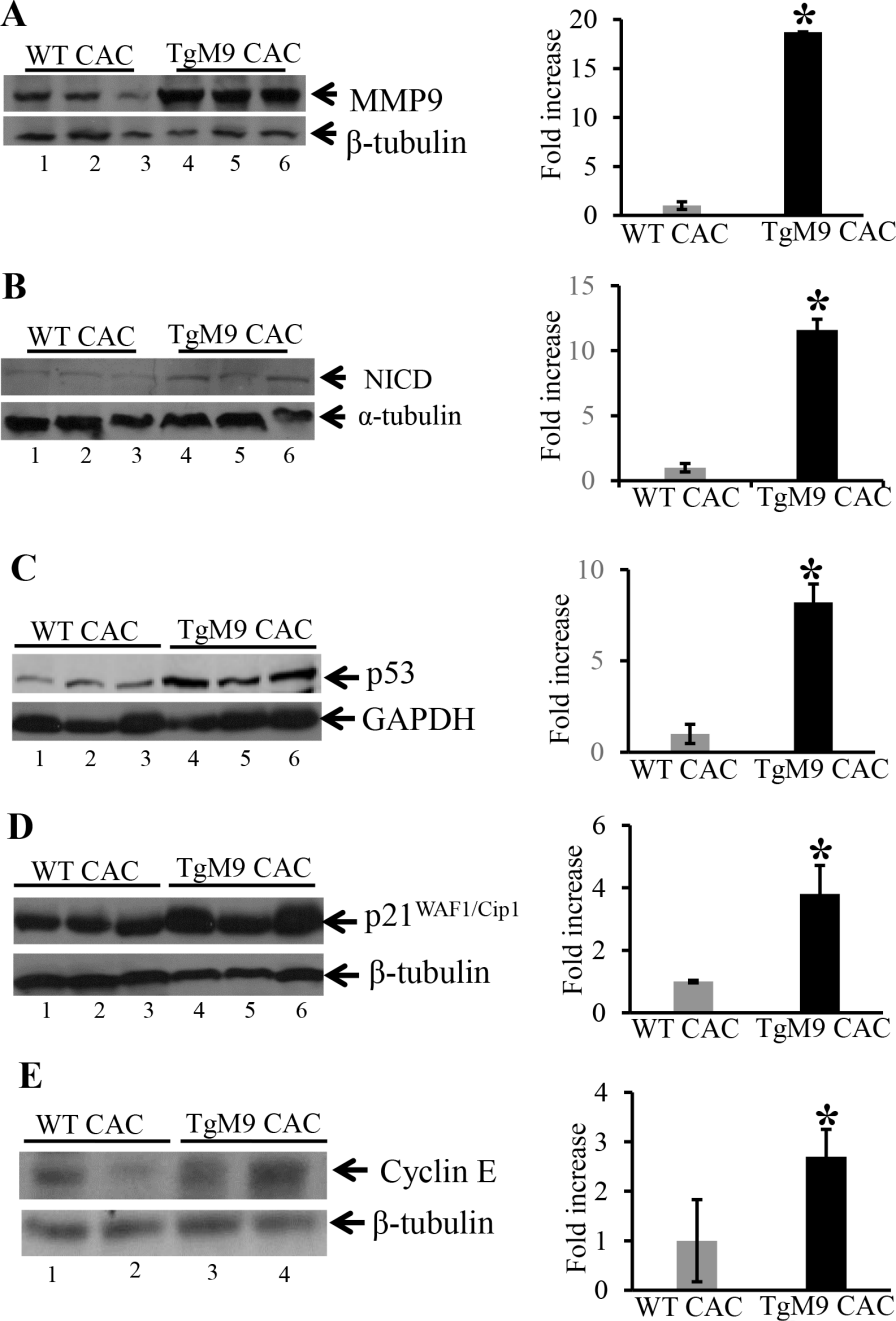
TgM9 mice exhibited altered protein expression of NICD, p53, p21 ^WAF1/Cip1^ and Cyclin E WBs of protein (25 μg/lane) from mucosal stripping of the TgM9 and WTs with CAC were performed and probed with (**A**) anti-MMP9; (**B**) anti-NICD; (**C**) anti-p53; (**D**) anti-p21^WAF1/Cip1^ and (**E**) anti-Cyclin E. The loading control for each blot was β-tubulin or GAPDH. Each blot was a representation of three individual experiments. Bar graphs are the representation of densitometry evaluations of the western blots. Each bar represents mean ± S.E., *p < 0.05.

Our next step was to dissect the mechanistic pathway by which epithelial derived-MMP9 mediates protection by exhibiting increased apoptosis in CAC. We have previously shown that MMP9 activates Notch1 signaling [16]. We hypothesize that MMP9 being a secretory proteinase cleaves transcellular protein Notch1. The cleaved cytoplasmic domain of Notch1 or Notch1 intracellular domain (called active Notch1/NICD) then translocate to the nucleus and activates ARF-p53-downstream targets. Therefore we assessed the activation of Notch1 in TgM9 mice. It is a well-recognized fact that p53 is the most common tumor suppressor gene which is mutated in almost all kinds of cancers [17]. Further, p53 mutations are the first ones to occur in CAC [18]. Figure 3A confirms the authenticity of constitutive expression of MMP9 in our TgM9 mice model (lanes 4–6) compared to WT littermates (lanes 1–3). There was also a significant increase in the protein expression of NICD among TgM9 mice (Figure 3B, lanes 4–6) compared to their WT littermates (Figure 3B, 1–3) in CAC. Figure 3C showed significantly increased protein levels of p53 among TgM9 mice (lanes 4–6) compared to WTs (lanes 1–3). p21^WAF1/Cip1^ is a well-documented protein involved in cell cycle regulation and is also a downstream target of p53 [19]. Figure 3D shows that there was a significant increase in the protein expression of p21^WAF1/Cip1^ among TgM9 mice (lanes 4–6) compared to their WT littermates (lanes 1–3) in CAC. Cyclin E, which is a cell cycle regulatory protein and forms a complex with p21^WAF1/Cip1^ [19] was also observed to be significantly higher among TgM9 mice (Figure 3E, lanes 4–6) compared to WTs (Figure 3E, lanes 1–3). Each blot was normalized for loading by immunoblotting with housekeeping genes- β-tubulin or GAPDH. Supplementary Figure S1 represents the protein expressions of MMP9, NICD, p53, Bax1, p21 ^WAF1/Cip1^ and Cyclin E among TgM9 compared to WT both treated/given water only (as a control group without CAC). These results together suggest that in CAC, epithelial derived-MMP9 is associated with activation of p53 via Notch1. p53 activation may lead to caspase-3 dependent apoptotic pathway and cell cycle cell cycle arrest via activation of its downstream target p21^WAF1/Cip1^ .

### Re-expression of MMP9 in MMP9^−/−^ mouse embryonic fibroblasts (MEFs) resulted in increased expression of NICD, p53, *p21WAF1/Cip1*, Bax1 and Cyclin A

**Figure 4:**
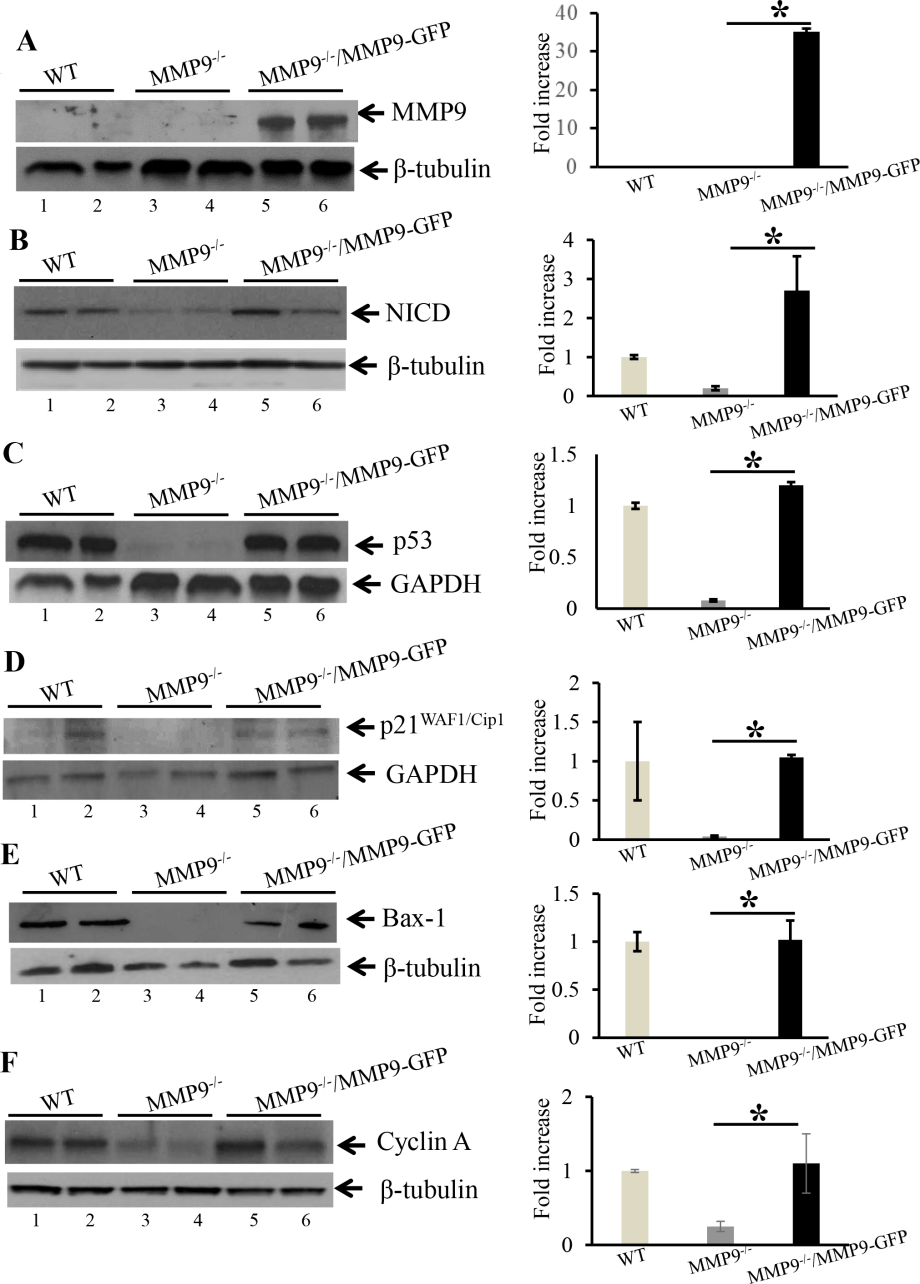
Re-erexpression of MMP9 in MMP9^−/−^ MEFs resulted in increased expression of NICD, p53 and p21^WAF1/Cip1^ WBs of protein (25 μg/lane) from whole cell lysate of stably transfected MMP9−/− MEFs with and without pEGFP-MMP9 plasmid were performed and probed with (**A**) anti-MMP9; (**B**) anti-NICD; (**C**) anti-p53; (**D**); anti-p21WAF1/Cip1 (**E**) anti-Bax-1; and (**F**) anti-Cyclin A. The loading control for each blot was β-tubulin or GAPDH. Each blot was a representation of three individual experiments. Each blot was a representation of three individual experiments. Bar graphs are the representation of densitometry evaluations of the western blots. Each bar represents mean ± S.E., *p < 0.05.

As a “proof of principle” model, to support our hypothesis that MMP9 plays a protective role in CAC and activates p53 resulting in increased apoptosis and cell cycle arrest, MMP9^−/−^ MEFs stably transfected with and without MMP9-GFP plasmids were used and results were compared with WT MEFs. Figure 4A shows the transfection efficiency of the MMP9-GFP plasmid into the MMP9^−/−^ MEFs cells by WB, indicating a significant increase in MMP9 expression (lanes 5–6, slightly higher band due to pEGFP plasmid) compared to vector control (lanes 3–4) and similar to WT MEFs (lanes 1–2). Figure 4B, 4C and 4D displays that re- rexpression of MMP9 in MMP9^−/−^/MMP9-GFP MEFs (MMP9^+/+^) was associated with significantly increased expression of NICD, p53 and p21^WAF1/Cip1^ (lanes 4–6) compared to vector control (lanes 3–4) and similar to WT MEFs (lanes 1–2) respectively. Figure 4E and 4F shows that MMP9 overexpression in MMP9^−/−^/MMP9-GFP MEFs (MMP9^+/+^) was also associated with significantly increased expressions of, pro-apoptotic factor Bax-1 and cell cycle protein Cyclin A (lanes 4–6) compared to vector control (lanes 3–4) and similar to WT MEFs (lanes 1–2) respectively. Each blot was normalized for loading by immunoblotting with housekeeping genes- β tubulin or GAPDH. Taken together, our *in vitro* data using stably transfected MMP9^−/−^ MEF cell line with and without MMP9 indicate that MMP9 activates p53 and p21^WAF1/Cip1^ via Notch1 resulting in increased apoptosis and cell cycle arrest respectively.

### Overexpression of MMP9 in human colon carcinoma cell line HCT116 displayed decreased cell proliferation and cell cycle arrest in S phase of cell cycle

**Figure 5:**
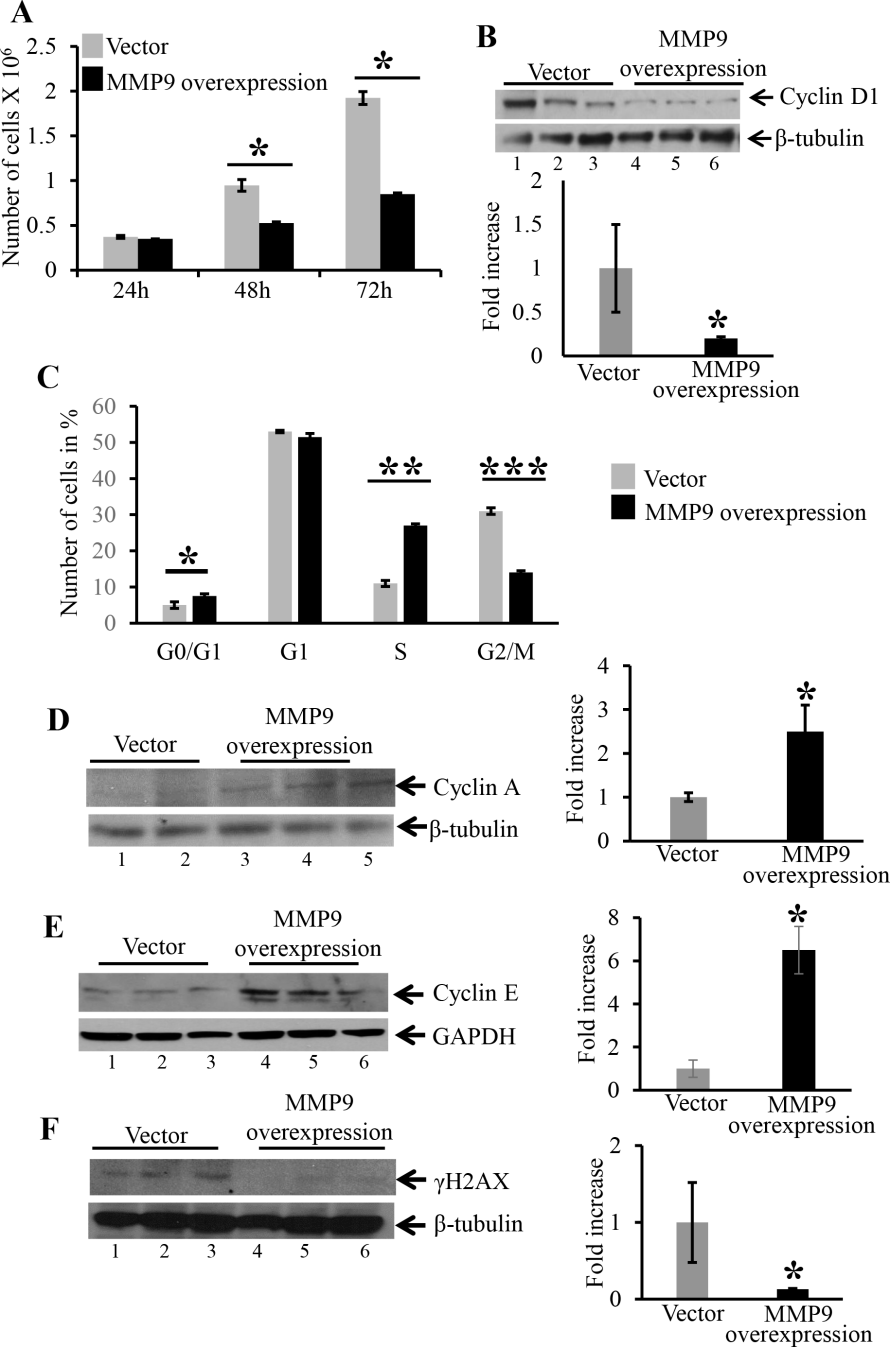
Overexpression MMP9 in human colon carcinoma cell line HCT116 displayed decreased cell proliferation, initiates cell cycle arrest in S phase and decreased DNA damage (**A**) The bar graph presentation of the number of stably transfected HCT116 cells with MMP9 (blue) and without MMP9/vector (grey) at 24, 48 and 72 hours respectively. Each bar represents mean ± S.E., *p < 0.005. (**B**) WB of protein (25 μg/lane) from whole cell lysates of stably transfected HCT116 cells with and without MMP9 were performed and probed with anti-Cyclin D1. (**C**) The bar graph presentation of FACS data showing number of cells in different phases of cell cycle- G0/G1, G1, S and G2/M of stably transfected HCT116 cells overexpressing MMP9 compared to vector control. Each bar represents mean ± S.E., **p < 0.0003 and ***p < 0.0005. WBs of protein (25 μg/lane) from whole cell lysate of stably transfected HCT116 cells with and without MMP9 were performed and probed with; (**D**) anti-Cyclin A; (**E**) anti-Cyclin E; and (**F**) anti-γH2AX. The loading control for each blot was β-tubulin or GAPDH. Each blot was a representation of three individual experiments. Each blot was a representation of three individual experiments. Bar graphs are the representation of densitometry evaluations of the western blots. Each bar represents mean ± S.E., *p < 0.05.

As another mechanistic approach to assess cell proliferation, we determined the role of MMP9 in cell cycle regulation. We used stably transfected colon carcinoma human cell line HCT116 with and without MMP9 [10] without γ-radiation. As described in ‘Material and Methods section’ equal number of HCT116 cells stably transfected with a pEGFP plasmid with or without the MMP9 gene were seeded in T25 cm^2^ flask and were counted after 24 h, 48 h and 72 h. Figure 5A is the bar graph presentation of the number of stably transfected HCT116 cells with and without MMP9, indicating that HCT116 cells overexpressing MMP9 (as indicated by blue bars) had significantly lower number of proliferating cells compared to the vector control at 48 and 72 hours (as indicated by grey bars). Figure 5B indicates that overexpression of MMP9 is associated with significantly lower levels of Cyclin D1 (another marker of cell proliferation [20–21]) (lanes 4–6) compared to vector control (lanes 1–3) respectively. Cyclins are the proteins that are involved in regulating cell cycle progression [22–23]. To recognize the link between MMP9 overexpression and cell cycle progression, HCT116 cells with and without MMP9 were stained with propidium iodide and were analyzed for different phases (G0/G1, G1, S and G2/M) by fluorescence activated cell sorting (FACS) (as described in Material and Methods section) technique. Figure 5C displays the bar graph presentation of the percentile of cells in each phase of the cell cycle indicating that HCT116 cells overexpressing MMP9 had a significant cell cycle arrest (28% ± 0.71) in S phase compared to vector (28% ± 0.83) resulting fewer (16% ± 0.71) MMP9 overexpressing cells reaching to G2/M phase compared to vector (29% ± 0.88). FACS analysis of G0/G1 phase indicated a significant increase in apoptotic cells among MMP9 overexpressing cells (8% ± 0.62) compared to vector control (5% ± 0.92). The lower number of apoptotic cells was due to the fact that HCT116 cell line being a carcinoma cell line is apoptosis resistant and requires γ-radiation (12 Gy) to induce apoptosis [10]. Figure 5D and 5E shows that MMP9 overexpression is associated with significant increase in protein levels of Cyclin A (lanes 4–6) and Cyclin E (lanes 4–6) compared to vector control (lanes 1–3) respectively. Each blot was normalized for loading by immunoblotting with housekeeping genes- β tubulin or GAPDH. Histone H2AX, a variant form of histone H2A, undergoes phosphorylationon serine 139 of its C terminal tail in response to DNA damage [24] as an early cellular response. Figure 5F displays significant decrease in DNA damage as indicated by decreased expression of γH2AX among MMP9 overexpressing cells (lanes 4–6) compared to vector control (lanes 1–3). These data together, demonstrate that MMP9 regulates cell proliferation by initiating cell cycle arrest in S phase of the cell cycle, associated with increased expressions of Cyclin A and Cyclin E and also protects from DNA damage.

### Attenuation of MMP9 in MMP9^−/−^ mice is associated with decreased expression of p19ARF an upstream regulatory molecule of wild type/non-mutated p53

**Figure 6:**
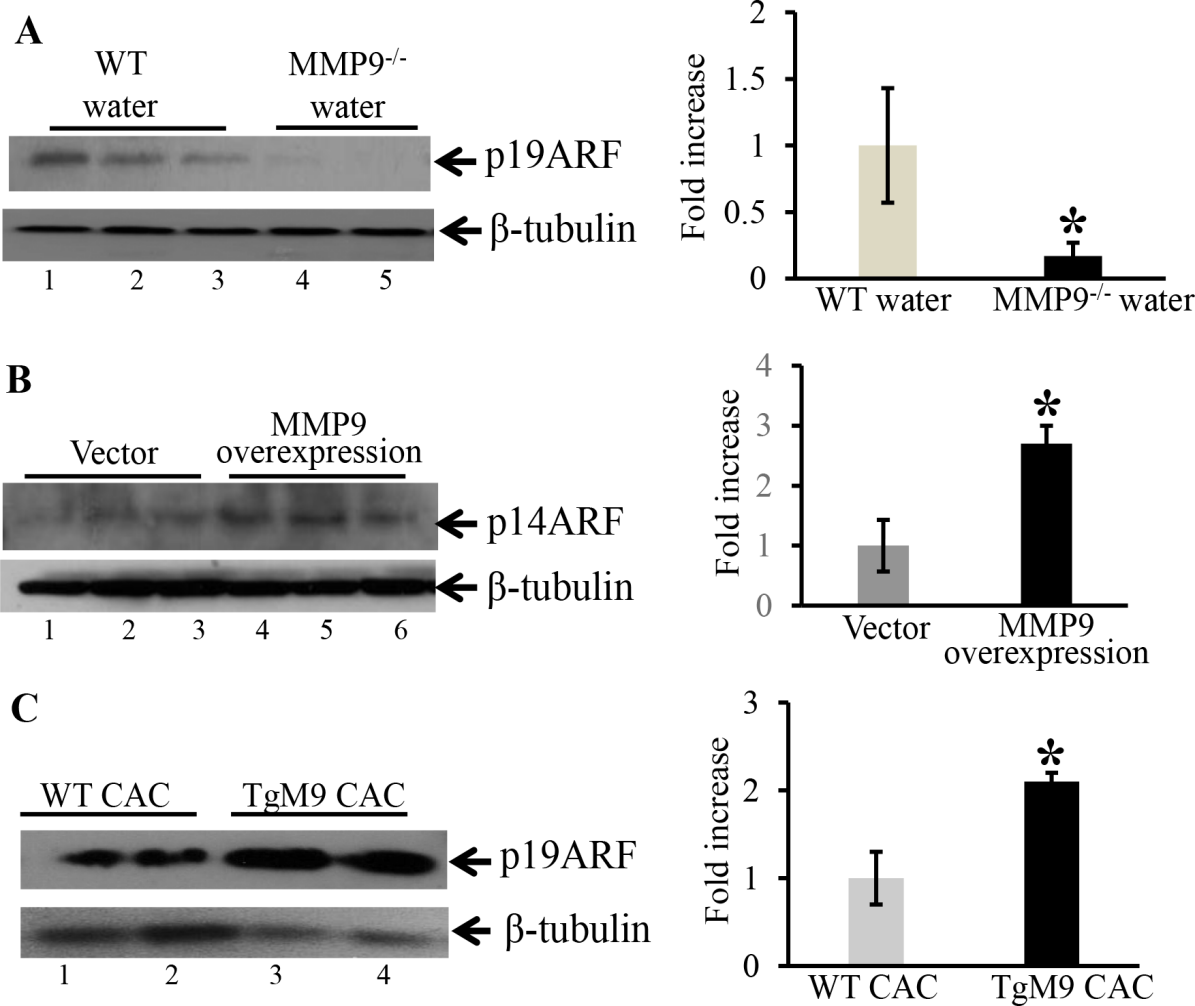
MMP9^−/−^ mice showed decreased expression of p19ARF an upstream regulatory molecule of p53 WB of (30 μg/lane) proteins (**A**) from the mucosal stripping of the colons of WT and MMP9^−/−^ mice (*n* = 10 each group) probed with anti-p19ARF; (**B**) from HCT116 overexpressing MMP9 probed with anti-p14ARF (human homologue of p19ARF). The loading control for each blot was β-tubulin. Each blot was a representation of three individual experiments. Each blot was a representation of three individual experiments. Bar graphs are the representation of densitometry evaluations of the western blots. Each bar represents mean ± S.E., *p < 0.05. (**C**) from the mucosal strippings of TgM9 and WT mice with CAC and probed with anti-p19ARF.

**Figure 7:**
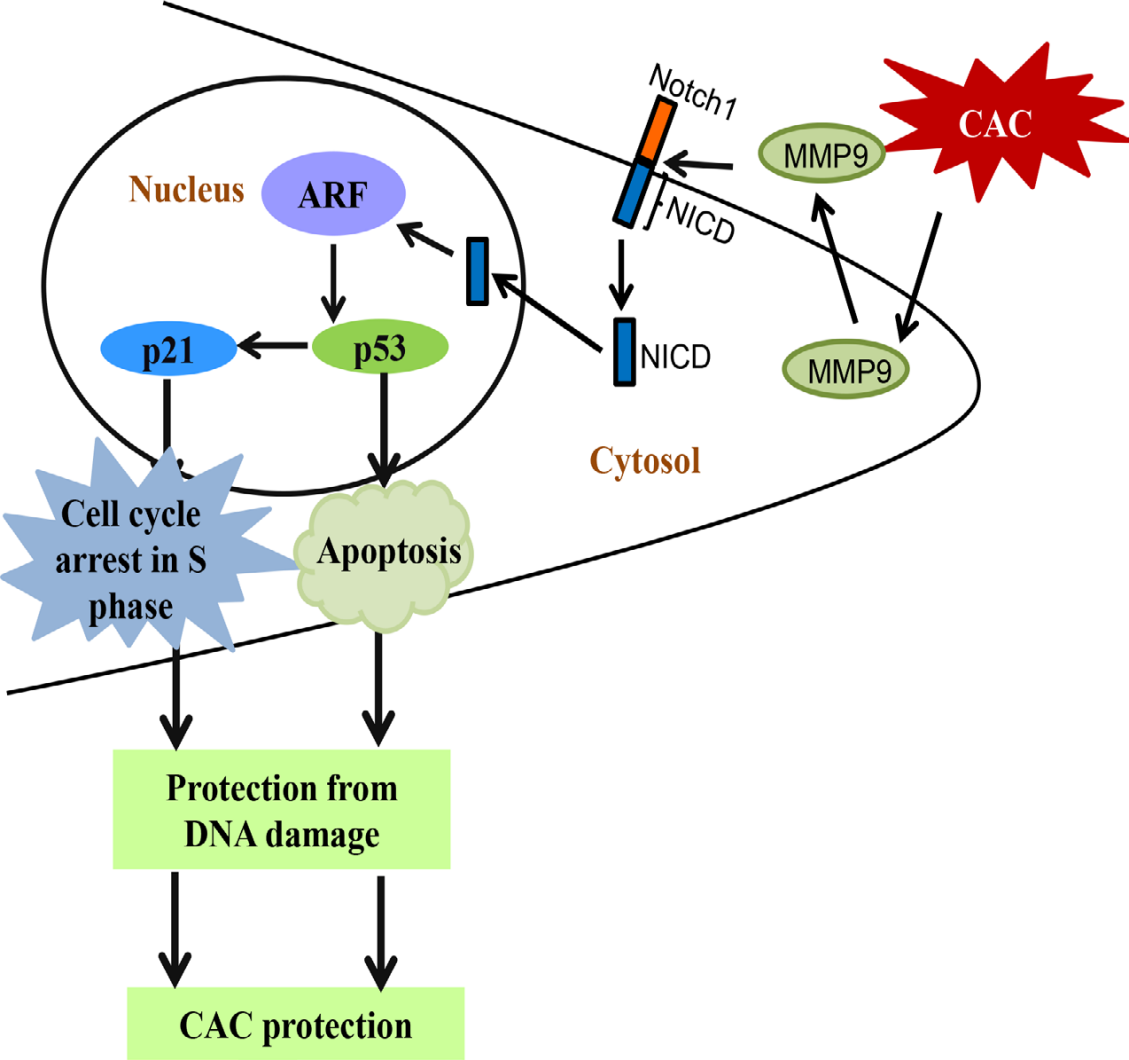
Schematic representation of “MMP9-Notch1-ARF-p53” mechanistic pathway of epithelial derived MMP9 mediated protection in CAC Abbreviations used are: NICD, Notch1 intracellular domain; ARF, alternative reading frame; CAC, colitis associated cancer.

It has been well documented that ARFs (alternative reading frame, termed p19ARF in mouse cells and p14ARF in human cells) and MDM2 (mouse double minute 2 homolog) are main regulatory proteins upstream of p53 and regulates its expression [25–27]. ARF binds to MDM2 and inhibits its binding with p53 by sequestering it to nucleus. Once sequestered in nucleus it cannot bind to p53 and cannot degrade p53 by ubiquitylation [28–29]. To identify the mechanistic link by which MMP9 activates wild type/non-mutated p53, we used WB analyses of upstream regulatory molecules of p53. We observed that at basal level MMP9^−/−^ mice (Figure 6A, lanes 4–5) compared to WT mice (Figure 6A, lanes-1–3) had significantly decreased protein expression of p19ARF. Figure 6B shows significantly increased expression of p14ARF (human homologue of p19ARF) among stably transfected HCT116 overexpressing MMP9 (lanes 4–6) compared to vector (lanes 1–3). However, we didn't observe any changes in the MDM2 expression by MMP9 (Supplementary Figure S2). We also observed that TgM9 mice (Figure 6C, lanes3–4) had significantly increased expression of p19ARF compared to WT littermates (Figure 6C, lanes 1–2) in CAC. These results suggest that the tumor suppressive role of epithelial derived-MMP9 is through a novel mechanistic pathway “MMP9-Notch1-ARF-p53 axis” in CAC (Figure 7).

## DISCUSSION

CAC is chronic inflammation driven carcinogenesis process, though the exact molecular mechanism by which chronic inflammation triggers CAC is still unexplored. Further, in CAC the physiological and pathological progression of inflammation to dysplasia and finally to carcinoma makes it tough to determine the rate limiting step for therapeutic strategies. It has been reported that CAC risk is up by 18–20% among UC patients, and even CD patients have shown a risk of developing CAC by 8%, and less than 50% of these die from CAC [3, 30] [31]. Therefore, identification and prognosis of disease risk genes in CAC has been a big hurdle for the efficacy of the therapeutic interventions in CAC.

Individual roles of MMPs with the initiation and perpetuation of intestinal inflammation as well as with the progression of CRC have long been cited in the literature. However, there are few sporadic studies discussing the precise role of MMPs in CAC [9], [11], [10], [3]. At the onset of inflammation, similar to the release of chemokines and cytokines, MMP9 is also active and highly up-regulated. Although its role has been well understood and documented in contexts of acute inflammation and CRC individually. However the precise role of MMP9 has never been studied in the setting of chronic colonic inflammation/CAC. We were the first ones to report the novel protective role of MMP9 in CAC [11] against its convention role of a mediator of acute inflammation. We have reported that in CAC, attenuation of MMP9 gene was associated with increased susceptibility to tumor incidence and tumor burden compared to WTs. We have reported that MMP9^−/−^ mice had significantly higher proliferation and lower apoptosis. We have also shown that MMP9 activates Notch1 signaling [16] and hence mediates protection in CAC [10]. This was supported by our findings that the inhibition of Notch1 signaling by the pharmacological blocker difluorophenacetly-L-alanyl-S-phenylglycine t-butyl ester (DAPT) is associated with a significant decrease in protein expressions of p53, p21 and cleaved caspase-3 [10]. We have also observed that inhibition of Notch1 signaling was associated with a significant decrease in ARF levels (Supplementary Figure S3). It has been known that both the epithelial cells and the neutrophil cells can secrete MMP9 in any tissue/organ. Therefore, to understand the precise mechanism of tumor suppressive role of MMP9 in CAC, it is very important to know which MMP9 ‘epithelial-derived’ or ‘neutrophil-derived’ is involved.

In the present study, we used TgM9 mice which can constitutively express MMP9 in colonic epithelium [15], to identify the role of epithelial derived MMP9 in CAC. We observed that constitutive expression of MMP9 in colonic epithelium is associated with lower tumor incidence, and retained crypt architecture compared to WT littermates, indicating that TgM9 mice were more protected from ulceration in CAC. In this study we have also shown that protection from CAC in TgM9 mice was associated with increased apoptosis compared to WTs. Our *in vivo* model delineated the underlying mechanism that epithelial derived-MMP9 mediates activation of p53 dependent caspase-3 apoptotic pathway as well as modulates the expressions of cell cycle regulatory proteins p21^WAF1/Cip1^ and Cyclin E via Notch1 signaling. Through our “proof of principle” model we verified the direct participation of MMP9 as a tumor suppressive protein, by using stably transfected MMP9^−/−^ MEFs with re-expression of MMP9. We could restore the protein expressions of NICD, p53, Bax-1, p21^WAF1/Cip1^ and Cyclin A (which otherwise were either absent or low in MMP9^−/−^ MEFs) by ‘knocking in *MMP9* gene’ in MMP9^−/−^ MEFs. Our *in vitro* model of human colonic carcinoma cell line HCT116 stably transfected with MMP9 displayed decreased proliferation compared to vector supporting the *in vivo* data of increased apoptosis. FACS analysis indicated cell cycle arrest in S phase among MMP9 overexpressing cells compared to vector control. We also observed increased expressions of cell cycle regulatory proteins (specifically associated with S phase of the cell cycle) Cyclin A and Cyclin E. The most striking and significant data in our eyes was the decrease in protein levels of γH2AX with MMP9 expression as indicated by our HCT116 *in vitro* model. These results together delineate the underlying molecular mechanism of the protective role of epithelial derived-MMP9 in CAC. Our *in vivo* and *in vitro* data showed direct correlation between MMP9 expression and ARF expression suggesting the mechanistic pathway by which MMP9 regulates p53 expression via Notch1 activation (Figure 7 and Supplementary Figure S3). Taken together the data suggest that epithelial derived-MMP9 acts as a tumor suppressor by activating MMP9-Notch1-ARF-p53 axis which results in increased apoptosis, initiates cell cycle arrest via activating p21^WAF1/Cip1^ as well as keep a check on DNA damage.

p53 is a well-established tumor suppressor that plays an important role in the development of CAC as well as sporadic colon cancers. p53^−/−^ mice are highly susceptible to CAC [32–33]. Cellular stress signals like DNA damage, UV light and oncogene activation trigger p53 activation and nuclear translocation. The activation of p53 modulates tumor suppression by initiating a transcriptional program that results in regulating apoptosis through caspase-3 activation or promoting cell cycle arrest or senescence. Among cell cycle regulatory proteins that are activated following cellular stress signals, the Cyclin-dependent kinase (CDK) inhibitor p21^WAF1/Cip1^ (also a downstream target of p53) plays essential roles in maintaining cellular integrity by inducing cell cycle arrest. Bimolecular complexes of CDKs and their Cyclin partners send the signals to responder molecules (e.g., p21^WAF1/Cip1^) to move the cell trough growth and division cycle [19]. Any damage to a cell's genome induces the activation of p21^WAF1/Cip1^, which thereby blocks the activity of Cyclin-CDK complex halting the cell cycle progression, until the damage has been repaired. Therefore the cell cycle does not progress and inadvertently stall the copying of damaged DNA sequences.

It has been well documented that ARF is the positive and MDM2 is the negative upstream regulatory proteins of p53. ARF functions as a tumor suppressor by directly binding and interfering with the p53-negative regulator MDM2, resulting in stabilization and activation of p53 [34]. It is worth to mention here that *p19ARF* sequences are intertwined with *p16INK4A* (inhibitor of the CDK4 and CDK6 kinases that plays a role in cell cycle arrest). The INK4a/ARF is the second most commonly altered gene locus in human cancer after p53 [35]. Since in our study we have observed cell cycle arrest in S phase and p16INK4A is responsible for G1/S phase cell cycle arrest. In the future, we would also explore the correlation between MMP9 and p16INK4A expressions [35]. This will help in understanding that the inclination of protection equilibrium is more towards apoptosis or is more for cell cycle arrest.

Genomic instability is critical for tumor progression. Uncontrolled endogenous DNA damage due to physiological cellular processes, chronic inflammation or exposure to carcinogens encourages faster growth of cancer cells over normal healthy cells by compromising regular cellular functions. Genomic instability is therefore directly associated with oncogene activation and inactivation of tumor suppressors [36]. Studies have suggested that γH2AX detection is an efficient biomarker in monitoring cancer progression [37]. Cancer cells typically have increased endogenous γH2AX levels compared to normal cells [37–38]. The downregulation of H2AX can be governed by the ARF/p53 pathway. Studies have shown that mutations in the ARF/p53 module, have elevated H2AX levels and exhibit accelerated growth [39–40]. Our study shows that in CAC, MMP9 protects from genotoxicity as indicated by decreased levels of γH2AX and acts a tumor suppressor.

MMP9 mRNA, protein level and activity are increased in human and animal models of CRC and potentiate colon cancer metastasis [41]. In general, metalloproteinase inhibitor as well as inhibitors of gelatinases, decrease colon cancer progression in animal models [42]. In mice, it has been reported that genetic ablation of MMP9 in APC^Min+/−^ mice resulted in 40% fewer tumors than littermate controls, although tumor size distribution remained unaffected [43]. On the other hand some studies have shown that in breast and colon cancer, MMP9 expression has been correlated with both increased and decreased survival and distant metastasis [44]. It is worthwhile to mention that *APCmin* mice represents sporadic cancer model which is different from CAC [45]. CAC represents ulceration due to chronic inflammation. However in recent years, new protective role of MMP9 has also been reported in CRC. It has been observed that MMP9 hemopexin domain has an inhibitory effect on migration and adhesion of colorectal carcinoma cells [46]. Another study has shown that reduction of plasma levels of MMP9 in either normal or integrin alpha1-null mice leads to decreased synthesis of angiostatin and consequent increased tumor growth and vascularization [47]. MMP9 exhibited protective role against lethal inflammatory mass lesions in mouse colon in absence of plasminogen [48] has also been documented. We hypothesize that irrespective of the tissue origin, epithelial derived- MMP9 will be protective in the setting of chronic inflammation. This is opposite to the function of neutrophil derived- MMP9. We also hypothesize that MMP9 (be epithelial-derived or neutrophil-derived) will always be a mediator of acute inflammation and sporadic cancers.

In very recent years protective functions of MMP9 have also been described in other cancers/malignancies. It has been reported that MMP9 promotes liver recovery from ischemia and reperfusion injury (IRI) by activating TGF-β and suggested that it plays dual roles (bad and good) in liver IRI, depending on the stage of the disease [49]. Protective roles of MMP9 have also been identified in oral cancers depending on the stage of the disease [50]. MMP9 displayed protective function in chronic kidney disease [51], lung cancer [52] and systemic autoimmune disease (lymphoproliferation and lupus) [53].

We have shown that in colon MMP9 activates Notch1 [16] whose signaling is important for cell fate determination, stem cell potential and lineage commitment and importantly in carcinogenesis. Notch1 function is highly context-dependent, and can either be oncogenic [54–56] or have a tumor suppressor function [57–60]. Different studies have shown a link between Notch1 regulation p53 activation, although clear cut association is yet to be established [61–63]. Once Notch1 signaling is active by releasing intracellular domain of the Notch receptor (NICD), it is then translocated to the nucleus, and forms a complex with transcription factor proteins forming a multiprotein complex, which directly activates transcription of downstream genes [62, 63]. We hypothesize that Notch1 may mediate tumor suppression by regulating p53 stability.

Our study therefore establishes the unique role of epithelial derived-MMP9 in CAC as a defensive molecule against its conventional role of a mediator of acute inflammation. We have also identified that activation of MMP9-Notch1-ARF-p53 axis is responsible for the two plausible mechanistic pathways- p53 dependent apoptosis or p21 mediated cell cycle arrest in mediating the protective role of epithelial derived-MMP9 and thereby controls or eliminates cells with damaged DNA. Our “proof of principle” model using MEFs with re-expression of MMP9 indicates that MMP9 driven signaling events MMP9-Notch1-ARF-p53 in CAC are unique and can evade the effect of the artefactual nature of the constitutive expression of MMP9. The outcome of this study highlights the paradox of using MMP9 inhibitors in current therapies to treat CAC patients and explains the failure of such treatments in the setting of chronic inflammation. Based on the traditional role of MMP9 as inflammation and CRC facilitator, humanized versions of MMP9 inhibitors are used as therapeutics for the CAC patients. However, efficiency of these inhibitors were tested either by acute colitis models or CRC models [64–65]. Unfortunately, none of them represent the CAC model. This implies a paradox in the current CAC treatments. Defining the protective role of MMP9 in CAC provides an opportunity for our research to be extended into clinical practice to improve human health and healthcare cost. Our study also emphasize that the use of humanized versions of MMP9 inhibitors or MMP9 neutralizing antibodies should be avoided for CAC patients.

## MATERIALS AND METHODS

### Animal models

All animal procedures were in compliance with the Guide for the Care of Use of Laboratory Animals from the US Public Health Service and with approval from the Animal Care Committee of Georgia State University. As described previously [15], 10 weeks old gender matched TgM9 and their wild-type (WT) littermates obtained while crossing the hetz of TgM9 of C57/B6 background were used for the study at the start of the experimental protocol. Extensive characterization of TgM9 has been published previously by us [66] and some new data has been added in Supplementary Figure S4. As described previously [13] [11], 10 weeks old gender matched MMP9^−/−^ and WT mice of C57/B6 background were also used for the study. They were maintained on a 12-hour dark-light cycle and allowed free access to nonpurified diet pellets and tap water.

### Colitis associated cancer induction

Both TgM9 (*n* = 20) and WT (*n* = 18) were divided into two groups- one group was induced with CAC and another group was given water only. The CAC group of TgM9 (*n* = 20) and WT (*n* = 18) were exposed to 3% DSS (w/v) (MP Biomedicals, Salon, OH) by oral administration through their drinking water *ad libitum* for 7 days. On day 8, their water was changed to regular drinking water. On day 21, their drinking water was changed back to 3% DSS for the second cycle of a week. This was followed by two weeks of recovery period with regular drinking water. On day 85, the mice were sacrificed after 3rd cycle of DSS and recovery. Colonscopy images (Xenon Nova 475, STORZ, Tuttlingen, Germany) were taken as well as colons being opened longitudinally to count the number of polyps and dysplastic lesions. We monitored body weight, stool consistency, and stool occult blood of all the mice during DSS and recovery cycles.

### H&E staining

Swiss rolls of the colon of mice induced with CAC were collected and H&E staining was performed. H&E stained sections were used to calculate the histological score based on crypt damage, infiltration of neutrophils, and foci of ulceration in the analyzed colons [67, 68].

### TUNEL staining

As described previously [15], paraffin sections of colons were deparaffinized and stained for TUNEL. Quantification of apoptosis was performed by counting number of apoptotic cells per crypt divided by total number of epithelial cells in the same crypt and was expressed as percentage. For each mice in each group 12 crypt were counted.

### Protein extraction and western blot analysis

As described previously [10] mucosal stripping were obtained from the TgM9 and WT mice (*n* = 20 per group) with and without CAC after the sacrifice for WB analysis. The antibodies used were anti-MMP9 (Abcam, Cambridge, MA), anti-NICD (Abcam), anti-caspase-3 (Cell Signaling, Beverly, MA) anti-p53 (Cell Signaling), anti-p21^WAF1/Cip1^ (BD Bioscience, San Jose, CA), anti-cyclin D1 (Santa Cruz, Dallas, TX), anti-Cyclin A (Cell Signaling), anti-Cyclin E1 (Santa Cruz), anti-γH2AX (Abcam), anti-p19ARF (Abcam), and anti-p14ARF (Abcam). Goat anti-mouse secondary antibody (1:2000; Bio-Rad, Hercules, CA) or goat anti-rabbit secondary antibody (1:2000, Bio-Rad) were used. Densitometry graphs were generated by using image acquisition and analysis software by VisionWorksLS Analysis Software (UVP, Upland, CA)

### Cell culture and transfection

As described previously [10], MEF cells, obtained from MMP9^−/−^ and WT mice. As describe previously stably transfected HCT116 cell lines (expressing wild type p53, a generous gift from Dr. VW Yang, Stony Brook University, NY) with and without MMP9 [10] were used to analyze the cell proliferation assay and cell cycle arrest. They were transfected for 72 h with a pEGFP plasmid with and without the MMP9 gene in 6 well plate. The transfected clones were selected under an antibiotic (Geneticin; GIBCO, Grand Island, NY). These transfected clones were screened for MMP9 expression and the three highest MMP9 expressing clones were selected for HCT116 cell line and two highest MMP9 expressing clones were selected for MEFs, and were sorted via flow cytometry (BD Biosciences).

### Cell proliferation assay

HCT116 cells were seeded in T25 cm^2^ flasks at the density 10^5^ per mL medium. After 24 h, 48 h and 72 h incubations, cells were counted by automated cell counter (Countess, Invitrogen, Grand Island, NY). Each experiment was done in triplicates.

### Cell cycle analysis

As described previously [69], flow cytometric (FACS) evaluation of cell cycle status of ~70% sub-confluent stably transfected HCT116 cell line overexpressing MMP9 and vector control grown in six well plates were stained by propidium iodide (Life Technologies; Grand Island, NY) (0.1% with 0.6% Triton X-100 in PBS) using FlowJo software (TreeStar, Ashland, OR).

### Statistical analysis

As described previously [15], data are presented as means ± SE. Groups were compared by Student's *t-test*. *P* values < 0.05 was considered statistically significant.

## SUPPLEMENTARY MATERIALS


