# Supplementary material for: Epithelial derived-matrix metalloproteinase (MMP9) exhibits a novel defensive role of tumor suppressor in colitis associated cancer by activating MMP9-Notch1-ARF-p53 axis
Source: Oncotarget. 2016 Nov 16;8(1):364–78. doi: 10.18632/oncotarget.13406 (PMC5352126; doi:10.18632/oncotarget.13406)
Supplement: Supplementary file 1 [file oncotarget-08-364-s001.pdf]

# Epithelial derived-matrix metalloproteinase (MMP9) exhibits a novel defensive role of tumor suppressor in colitis associated cancer by activating MMP9-Notch1-ARF-p53 axis

## Supplementary Materials

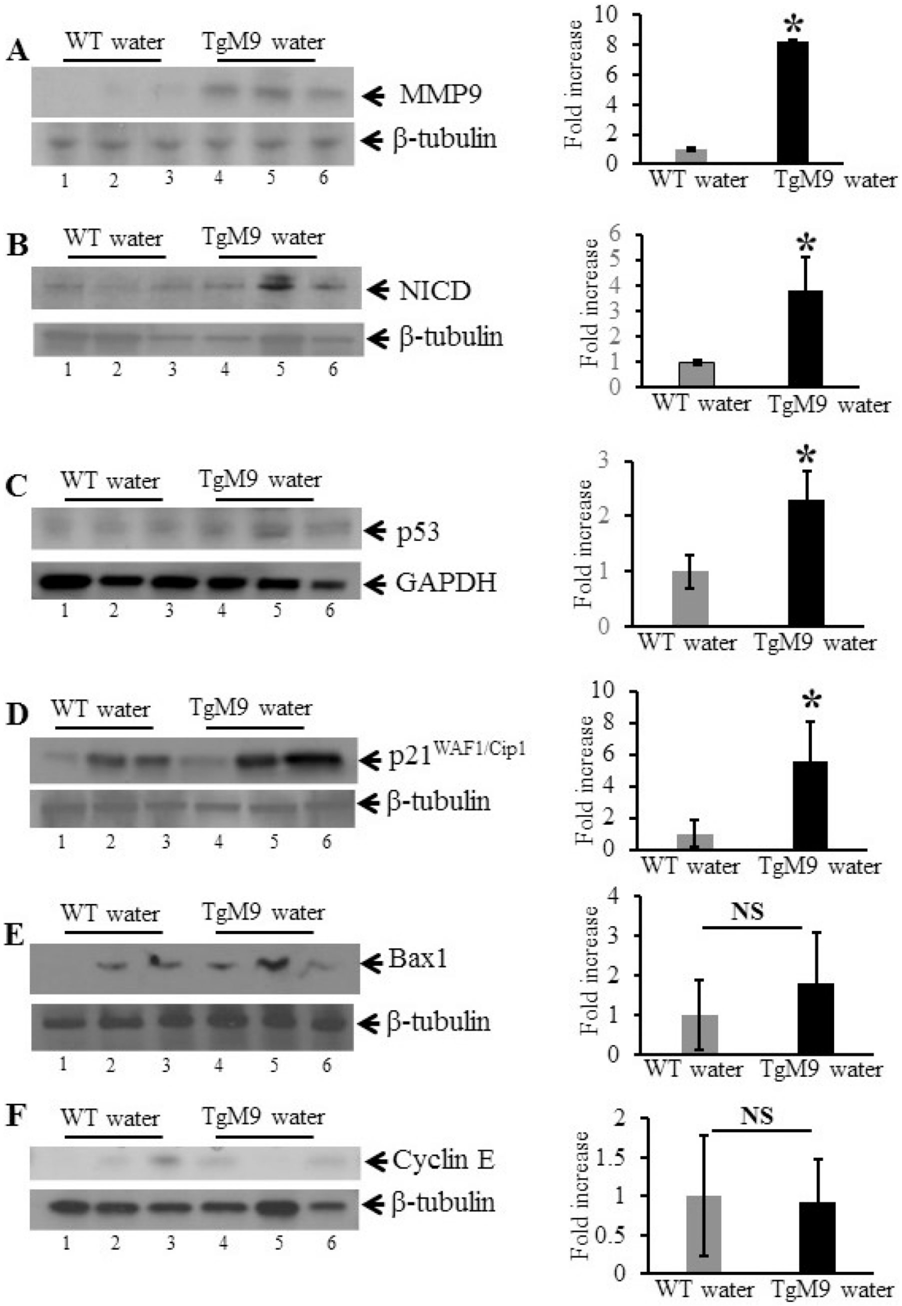


**Supplementary Figure S1: Protein expressions of NICD, p53, caspase-3, p21$^{WAF1/Cip1}$ and Cyclin E in TgM9 mice and WTs without CAC.** Western blot of proteins (30 μg/lane) from the mucosal stripping of the colons of TgM9 and WT mice ($n$ = 10 each group) probed with (**A**) anti-MMP9; (**B**) anti-NICD; (**C**) anti-p53; (**D**) anti-p21$^{WAF1/Cip1}$; (**E**) Bax1 and (**F**) anti-cyclin E. Bar graphs are the representation of densitometry evaluations of the western blots. Values are representative of two experiments.

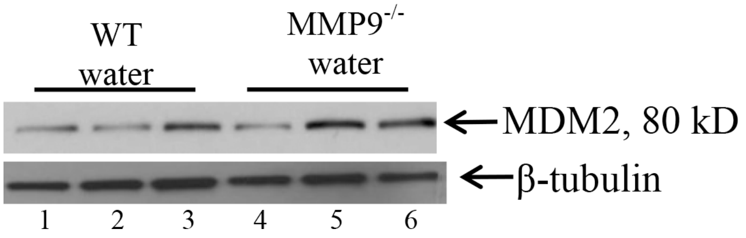


**Supplementary Figure S2: Expression of MDM2 an upstream regulatory molecule of p53 was MMP9 independent.** Western blot of proteins (30 µg/lane) from the mucosal stripping of the colons of WT and MMP9$^{-/-}$ mice (*n* = 10 each group) probed with anti-Mdm2. Values are representative of three experiments.

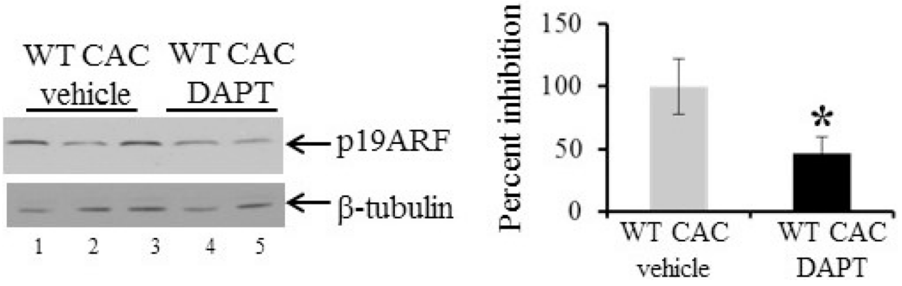


**Supplementary Figure S3: Inhibition of Notch1 signaling is associated with decreased levels of p19ARF.** WT mice (*n* = 18 each group) were induced CAC and were treated with i.p. of difluorophenacetly-L-alanyl-S-phenylglycine t-butyl ester (DAPT) or vehicle during each DSS cycle for 5 consecutive days. Western blot of proteins (30 µg/lane) from the mucosal stripping of the colons were probed with anti-p19ARF. Western blot was quantitated by scanning densitometry. Values are representative of two experiments, each bar represents mean ± S.E., *$p < 0.05$.

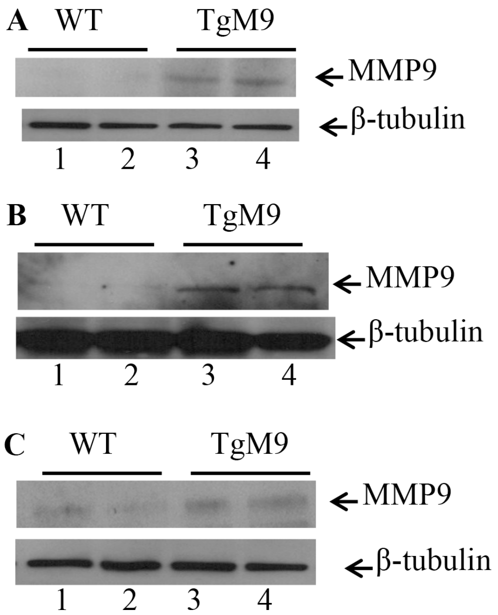


**Supplementary Figure S4: Increased expression of MMP9 in other organs having villin expression.** Western blot of proteins (30 µg/lane) from the tissues lysates (*n* = 6 each group) probed with anti-MMP9 displayed increased expression of MMP9 among TgM9 mice (lanes 3–4) in (**A**) ileum; (**B**) kidney; and (**C**) spleen compared to WT littermates (lanes 1–2) at basal level. Values are representative of three experiments.
